# Metamaterial‐Assisted Illumination Nanoscopy with Exceptional Axial Resolution

**DOI:** 10.1002/advs.202404883

**Published:** 2024-08-20

**Authors:** Yeon Ui Lee, Shilong Li, Junxiang Zhao, Clara Posner, Jin Zhang, Zhaowei Liu

**Affiliations:** ^1^ Department of Electrical and Computer Engineering University of California San Diego 9500 Gilman Drive La Jolla CA 92093 USA; ^2^ Department of Physics Chungbuk National University Cheongju Chungbuk 28644 South Korea; ^3^ Interdisciplinary Center for Quantum Information State Key Laboratory of Modern Optical Instrumentation College of Information Science and Electronic Engineering Zhejiang University Hangzhou 310027 China; ^4^ Department of Pharmacology University of California San Diego 9500 Gilman Drive La Jolla CA 92093 USA; ^5^ Materials Science and Engineering Program University of California San Diego 9500 Gilman Drive La Jolla CA 92093 USA

**Keywords:** bioimaging, metamaterials, nanometer‐scale axial localization, structured illumination microscopy, super‐resolution microscopy

## Abstract

Recent advancements in optical metamaterials have opened new possibilities in the exciting field of super‐resolution microscopies. The far‐field metamaterial‐assisted illumination nanoscopies (MAINs) have, very recently, enhanced the lateral resolution to one‐fifteenth of the optical wavelength. However, the axial localization accuracy of fluorophores in the MAINs remains rarely explored. Here, a MAIN with a nanometer‐scale axial localization accuracy is demonstrated by monitoring the distance‐dependent photobleaching dynamics of the fluorophores on top of an organic hyperbolic metamaterial (OHM) substrate under a wide‐field single‐objective microscope. With such a regular experimental configuration, 3D imaging of various biological samples with the resolution of ≈40 nm in the lateral dimensions and ≈5 nm in the axial dimension is realized. The demonstrated imaging modality enables the resolution of the 3D morphology of nanoscopic cellular structures with a significantly simplified experimental setup.

## Graphical abstract

It demonstrates that with an organic hyperbolic metamaterial substrate, both the lateral and axial imaging resolutions of a fluorophore can be improved by exploiting the metamaterial's hyperbolic dispersion and the resultant photobleaching‐dynamics variation of the fluorophore. The demonstrated far‐field metamaterial‐assisted illumination nanoscopy with a spatial resolution of ≈40 nm in the lateral dimensions and ≈5 nm in the axial dimension offers many exciting possibilities in biological research.

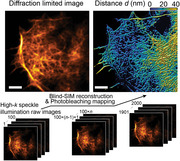

## 1 Introduction

Super‐resolution fluorescence microscopy, i.e., nanoscopy, has been widely used for examining the nanoscale structure and biological dynamics of living cells and tissues.^[^ ^1^ ^]^ It overcomes the classical resolution barrier in optical microscopy—i.e. 200–300 nm in the lateral plane and 500–800 nm along the axial direction^[^ ^2^ ^]^—by exploiting different physical mechanisms.^[^ ^3^ ^]^ To improve the lateral resolution most of the state‐of‐the‐art super‐resolution nanoscopies rely on modifying the temporal dynamics of fluorophores to perform the single‐molecule localization, such as photoactivated localization microscopy (PALM)^[^ ^4^ ^]^ and stochastic optical reconstruction microscopy (STORM),^[^ ^5^, ^6^ ^]^ or taking advantage of the nonlinear responses of fluorophores to engineer the point spread function, such as stimulated emission depletion microscopy (STED)^[^ ^7^ ^]^ and ground state depletion microscopy (GSD).^[^ ^8^ ^]^ Additionally, their axial resolution can readily be enhanced by 4Pi or I5M super‐resolution techniques.^[^ ^9^, ^10^ ^]^ In parallel with these fluorophore‐engineering‐based super‐resolution microscopies, structured illumination microscopy (SIM) has also been developed to obtain 3D super‐resolution images^[^ ^11^, ^12^ ^]^ by sampling the object with nonconventional illuminations. Nevertheless, each super‐resolution fluorescence microscopy has its own strengths and weaknesses that need to be considered for specific biological applications.

In a different context, engineered optical materials, known as electromagnetic metamaterials, have been investigated in a wide range of research fields including the super‐resolution microscopy.^[^ ^13^ ^]^ These metamaterials have provided unique insights into the way of surpassing the diffraction limit, e.g., the perfect lens made of negative‐refraction metamaterials allows for achieving an ideal image.^[^ ^14^ ^]^ Moreover, they offer exceptional capabilities to extend the resolution of existing super‐resolution techniques. For example, metamaterial‐assisted illumination nanoscopies (MAINs) such as plasmonic structured illumination microscopy (PSIM)^[^ ^15^ ^]^ and localized plasmonic structured illumination microscopy (LPSIM)^[^ ^16^, ^17^ ^]^ have improved the lateral resolution to ∼λ/5 and ∼λ/6, respectively, compared to the limit of ∼λ/4 in the classical SIM, where λ is the wavelength of illumination. Very recently, the lateral resolution has been further enhanced to ∼λ/15 by using either multilayered hyperbolic metamaterials (HMMs)^[^ ^18^, ^19^ ^]^ or organic hyperbolic metamaterials (OHMs).^[^ ^20^, ^21^ ^]^ While there has been significant success in improving the lateral resolution with the MAINs, accurately localizing fluorophores along the axial direction remains a major challenge.

In this work, we demonstrate a 3D MAIN with a spatial resolution of ≈40 nm in the lateral dimensions and ≈5 nm in the axial dimension. The 3D MAIN is implemented based on an OHM substrate with a wide‐field single‐objective speckle‐illumination microscope to extract the lateral super‐resolution information through frequency mixing and the axial super‐resolution information through the distance‐dependent photobleaching dynamics of fluorophores on top of the OHM. We verify the 3D super‐resolution capability by obtaining images of biological specimens. Compared with other 3D super‐resolution methods mentioned above, the demonstrated 3D MAIN features simple implementation, low phototoxicity, and no need for special fluorophores (see Table S1, Supporting Information for a comprehensive comparison). Therefore, such an organic metamaterial‐enabled super‐resolution imaging technique with the nanometer‐scale axial localization ability could open many exciting possibilities in biological research.

## 2 Results and Discussion

### 2.1 MAIN with Nanometer‐Scale Axial Localization Accuracy

**Figure 1:**
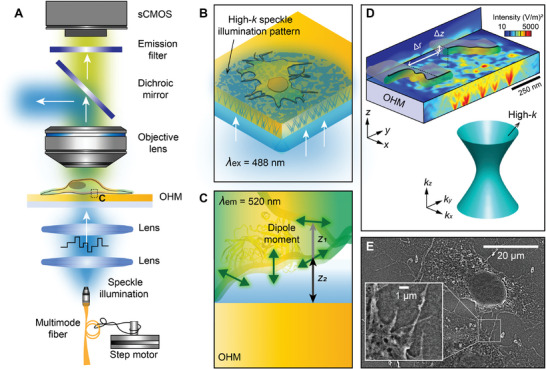
MAIN capable of nanometer‐scale axial localization. A) Schematic drawing of a wide‐field speckle‐illumination microscope used in 3D MAIN. A time‐varying speckle illumination was produced by shaking a multimode fiber with a step motor, and then directed to the stained bio‐sample through the underneath OHM substrate. The excited fluorescence signals were collected by an objective lens and imaged with a sCMOS camera. B) High‐*k* illumination enabled by the OHM. Optical modes with a high *k* in the OHM were excited by the incident speckle beam from its bottom surface, resulting in randomly distributed hot spots with a subwavelength volume on its top surface. A lateral super‐resolution image with such a random high‐*k* illumination was reconstructed based on the Blind SIM algorithm. C) Nanometer‐scale axial localization enabled by the OHM high‐*k* illumination. Since there is a one‐to‐one relationship between the distance—with respect to the OHM top surface—of a fluorophore and its photobleaching dynamics, the axial super‐resolution information was obtained via this distance‐photobleaching relationship based on a series of lateral super‐resolution images. D) 3D OHM MAIN. Randomly distributed near‐field high‐*k* speckles generated in the OHM transfer the fine structural information of fluorophores—e.g. the lateral (Δ*r*) and axial (Δ*z*) separations of two fluorophores—into the far‐field microscope. E) Typical scanning electron microscope (SEM) image of a bio‐sample (Cos‐7 cells) prepared on top of an OHM substrate.

The 3D MAIN setup is illustrated in **Figure** **1**A. A transmission speckle‐illumination fluorescence microscope system was used in this work, which was also used in the previously reported MAINs^[^ ^18^, ^21^ ^]^ (see Experimental Section for details). By employing a step motor to stretch the input fiber coupled with a 488‐nm laser, various speckle patterns were generated on the backside of an OHM substrate. High spatial frequency (high‐*k*) speckle illuminations on the top surface of the OHM substrate were then created due to coherent superposition of the wavelets resulting from roughness‐ and/or particulate‐induced scattering when the input laser was passing through the OHM (Figure 1B). These dynamically controllable high‐*k* near‐field speckles provided ultra‐fine random sampling of the object and thus allowed for high lateral resolution beyond the diffraction limit. It is worth noting that the presence of the OHM substrate also enhanced the photostability of fluorophores situated on top of it due to the Purcell effect.^[^ ^22^, ^23^, ^24^ ^]^ Emission signals from the fluorophores were collected by a 40×/0.6‐NA objective lens, and acquired by a scientific complementary metal‐oxide‐semiconductor (sCMOS) camera after a band‐pass filter (520 ± 20 nm). The axial super‐resolution information was obtained through the distance‐dependent photobleaching decay rate^[^ ^23^ ^]^ (Figure 1C).

To have a better physical intuition about the 3D MAIN, finite‐difference time‐domain (FDTD) simulations (see Experimental Section for details) were carried out. Figure 1D shows the simulated electric field intensity distribution of the illuminating speckles; the speckle result on top of a glass substrate is given in Figure S1 (Supporting Information). Compared to the conventional glass substrate, the OHM substrate is able to confine the input light into deep subwavelength volumes, which contributes to the high‐*k* frequency mixing and thus the high lateral resolution. Such a strong light confinement ability of the OHM is due to its hyperbolic dispersion,^[^ ^20^, ^21^ ^]^ as shown in the inset of Figure 1D. In addition, the hyperbolic dispersion of the OHM leads to an exceptionally high Purcell factor,^[^ ^22^, ^23^, ^24^ ^]^ which is the key factor for determining the axial position of fluorophores. The combination of the OHM's strong light confinement and Purcell effect presents a unique opportunity to resolve deep subwavelength features of fluorophores in 3D.

### 2.2 Steps to Obtain 3D Super‐Resolution Image

**Figure 2:**
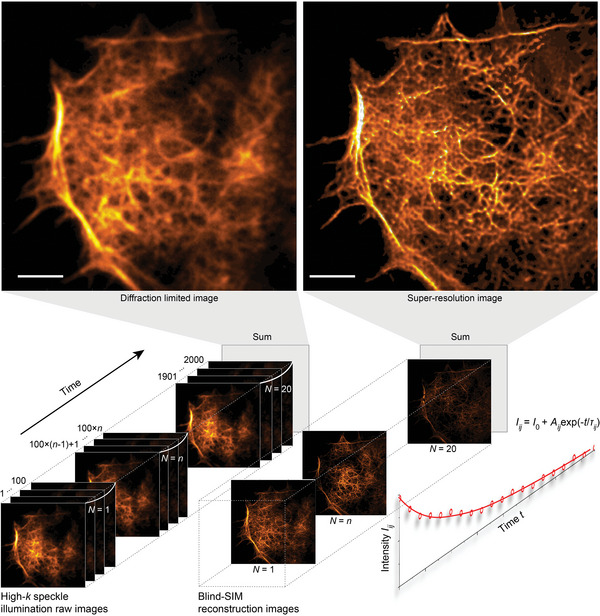
Procedure flow to extract both the lateral and axial super‐resolution information in 3D MAIN. First, 2000 diffraction‐limited images are recorded with an exposure time of 200 ms. Then, *N*  =  20 lateral super‐resolution images are reconstructed from every *L*  =  100 successively diffraction‐limited images using the Blind‐SIM algorithm. Owing to the photobleaching, the emission intensity of the fluorophores drops exponentially over time; the axial super‐resolution information is then obtained by fitting the intensity decay curve *I_ij_* for each pixel (*i*, *j*) of the 20 reconstructed lateral super‐resolution images. Scale bars: 5 µm.

The 3D MAIN is applied to obtain 3D super‐resolution images of the morphology in close contact areas, e.g. focal adhesions or filopodia, for biological samples attached to OHM substrates, and the procedure flow is summarized in **Figure** **2**. Cos‐7 cells (Figure 1E) were grown on top of the OHM substrates and transiently transfected with a plasma membrane or actin localized fluorescent protein (see Figure S2, Supporting Information for details). Using the 3D MAIN system, 2000 different diffraction‐limited images of the cells were recorded with spatially varied speckle illuminations. The Blind‐SIM reconstruction^[^ ^25^ ^]^ was then performed sequentially every 100 frames to obtain 20 super‐resolution images (≈40 nm in the lateral resolution). With this time series of super‐resolution images, the photobleaching lifetime of fluorophores was obtained by fitting the respective intensity decay curves, pixel by pixel. Adding knowledge about the distance‐dependent photobleaching lifetime enabled the retrieval of the axial position of the fluorophores^[^ ^23^ ^]^ (see Figure S3, Supporting Information for details). In the end, a 3D super‐resolution morphology image of the cells was obtained. We provide an additional straightforward procedure flow of the 3D MAIN via numerical simulations (see Figure S4, Supporting Information for details).

### 2.3 3D Super‐Resolution Imaging of Cos‐7 Cells

**Figure 3:**
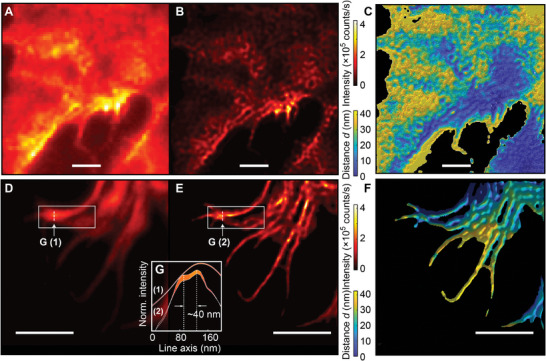
3D MAIN super‐resolution imaging of fluorescently labeled plasma membrane and actin of Cos‐7 cells. A,B) Conventional wide‐field fluorescence image A) and MAIN super‐lateral‐resolution image B) of the fluorescently labeled plasma membrane of a Cos‐7 cell. C) 3D MAIN super‐resolution image of the fluorescently labeled Cos‐7 cell membrane. Scale bars: 2 µm. D,E) Conventional wide‐field fluorescence image (D) and MAIN super‐lateral‐resolution image (E) of the fluorescently labeled actin of a Cos‐7 cell. F) 3D MAIN super‐resolution image of the fluorescently labeled actin of the Cos‐7 cell. Scale bars: 1 µm. G) Intensity profiles along the line ‘G (1)’ in (D) and the line ‘G (2)’ in (E), respectively. A ≈40‐nm center‐to‐center lateral separation of two intensity peaks is clearly visible.

**Figure** **3** summarizes the 3D MAIN super‐resolution imaging results for the fluorescently labeled plasma membrane and actin of Cos‐7 cells. Compared to the conventional wide‐field fluorescence images (Figure 3A,D) and the super‐lateral‐resolution images (Figure 3B,E), our 3D MAIN allows for the visualization of a 3D morphology of the cell samples beyond the diffraction limit over all spatial directions (Figure 3C,F). Therefore, the contact areas of the cell membrane and actin can be investigated with greater precision using the 3D MAIN.

The resolution of the obtained 3D MAIN images of the cell samples is measured by the lateral separation and the axial localization accuracy. Figure 3G shows a ≈40‐nm center‐to‐center lateral separation of two intensity peaks, which corresponds to a lateral resolution of ∼λ/13. In general, 3D MAIN uses a bi‐exponential function for the axial localization of fluorophores and only examines fluorophores in a distance range of less than 50 nm: In the photobleaching lifetime fitting process, the faster component results from the photobleaching of fluorophores located far away (*d* > 50 nm) from the OHM surface, while the fluorophores in the proximity of the OHM surface contribute to the slower decaying emissions (see Figure S3, Supporting Information for details).

### 2.4 Estimation of Axial Localization Accuracy

**Figure 4:**
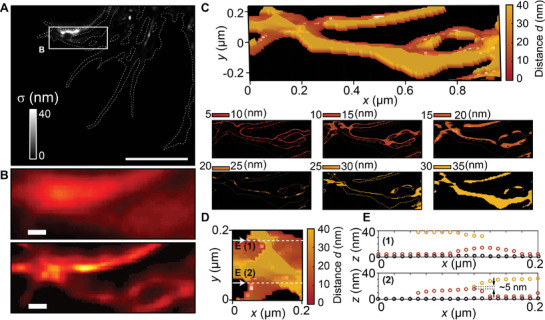
Estimation of axial localization accuracy in 3D MAIN. A) Distribution of standard errors of the mean σ of calculated distance value using a bi‐exponential fitting function for the 3D MAIN image of the fluorescently labeled actin in a Cos‐7 cell shown in Figure 3G. Scale bars: 1 µm. B) Conventional wide‐field fluorescence image (top panel) and MAIN super‐lateral‐resolution image (bottom panel) of the cell actin around the area with a large σ. Crossing of actins is visible in the MAIN image but how these actins overlap is unknown. Scale bars: 100 nm. C–E) 3D MAIN super‐resolution images (C,D) and height profile (E) of the actin around the large‐σ area using a tri‐exponential fitting function. The axial positions of the overlapped actins are resolved.

The axial localization accuracy of 3D MAIN is estimated via the standard errors of the mean (s.e.m., σ), which can be obtained during the intensity decay fitting process.^[^ ^23^ ^]^ **Figure** **4**A shows the σ distribution for the 3D MAIN image of the fluorescently labeled actin in a Cos‐7 cell (Figure 3F). As observed in our previous study,^[^ ^23^ ^]^ most regions have small σ(σ < 3 nm). However, there is a large σ(σ > 3 nm) in certain areas, which is the signature of actin overlapping (Figure 4B). In this case, multi‐exponential functions must be used to resolve the overlapped actins; for example, Figure 4C–E shows the 3D MAIN super‐resolution image of the cell actin around the large‐σ area with a tri‐exponential fitting function and now the two actins are clearly distinct with a ≈5‐nm axial separation and a localization accuracy below 3 nm.

For more intricate fluorophore distributions in complex cellular environments, more photobleaching processes must be considered. However, the resulting multi‐exponential fitting typically requires a much denser sampling and is known to be sensitive to noise. In the current 3D MAIN implementation, we applied the Blind‐SIM algorithm for image reconstruction which used 100 raw data frames for each super‐resolution image; with a total acquisition of 2000 images before the fluorophores completely bleach, 20 reconstructed intensities per pixel can be used for the photobleaching lifetime fitting. This is the main limitation to the multi‐exponential fitting as well as the axial localization accuracy. Recently, the use of deep neural networks (DNNs) for image reconstruction in super‐resolution microscopies has demonstrated superior performance in many challenging cases including reconstruction with limited numbers of input frames.^[^ ^26^, ^27^ ^]^ Future application of the DNN based image reconstruction could greatly reduce the required number of raw images for each super‐resolution frame, and correspondingly provide a much denser intensity decay sampling for a much more accurate multi‐exponential fitting.

To ensure accurate imaging, the motor shaking rate should be kept below 1 Hz to avoid image distortion, considering the 200‐ms exposure time and the motor startup and braking time. Additionally, the photobleaching rate should be maintained below 0.01 Hz to balance between the signal decay time and the total imaging time. The optimization of motor shaking rate, photobleaching rate, and imaging capture rate need to be done toward practical applications in future works.

## 3 Conclusion

In conclusion, we have demonstrated a 3D super‐resolution imaging technique, namely 3D MAIN, that reaches a lateral resolution down to ≈40 nm with an axial localization accuracy of ≈5 nm for biological imaging. By using an OHM substrate, the large‐spatial‐wavevector illumination overcomes the lateral resolution barrier, while the distance‐dependent (with respect to the OHM substrate) photobleaching dynamics of fluorophores enables high‐precision axial localization. The 3D MAIN provides a powerful tool to unveil the subwavelength scale cellular architectures and could significantly impact cell biology. We envision this nanoscopy technology could dramatically improve our understanding in many active research areas, for example, the membrane contact sites such as the endoplasmic reticulum‐plasma membrane contact sites, which play important roles in cell signaling.^[^ ^28^, ^29^ ^]^

## 4 Experimental Section

### Optical Set‐Up

An inverted fluorescence microscope (Olympus IX‐83) was used with a 488‐nm excitation laser (Coherent Genesis MX488‐1000 STM) coupled through a multimode fiber and a microscope condenser.^[^ ^18^ ^]^ A step motor was applied to mechanically shake the multimode fiber for generating the required random laser speckles. The speckle patterns at the fiber exit were imaged on the backside of an OHM‐coated microscope slide, projecting diffraction‐limited speckles. The speckle intensity onto the OHM was ≈50 W cm^−^^2^. These diffraction‐limited speckles were converted by the OHM substrate to sub‐diffraction‐limit speckles on top of the OHM, which were used to illuminate the fluorophores. The fluorescence signals were collected by a sCMOS camera (Hamamatsu Orca Flash 4.0 v3) after an emission filter (520 ± 20 nm).

### OHM Film Fabrication and Optical Characterization

A solution was prepared by dissolving 100 mg of >98% regioregular head‐to‐tail P3HT (rr‐P3HT) molecules (Sigma–Aldrich, average molecular weight ≈87,000 g mol^−1^) in 1 mL of chlorobenzene solvent, and then heated at 50 °C for 3 h. Subsequently, this rr‐P3HT solution was spin‐coated onto plasma‐cleaned glass substrates. The thickness of the produced rr‐P3HT OHM film was obtained based on variable angle spectroscopic ellipsometry (VASE) measurement, which was also used to determine the optical permittivity of the OHM film as previously reported.^[^ ^20^ ^]^ Cos‐7 cells were cultured on the OHM film substrate and transiently transfected (see Figure S2, Supporting Information for details).

### FDTD Simulation of High‐k Speckle Patterns

3D FDTD simulations were conducted (Ansys Lumerical FDTD). The experimentally obtained permittivity of the OHM was used in the simulations.^[^ ^20^, ^21^ ^]^ To calculate near‐field intensity distributions in the vicinity of the OHM‐coated glass, a 2D *xy*‐plane power monitor and a 2D *xz*‐plane power monitor were placed. The perfectly matched layers (PMLs) were used along the *x*, *y*, and *z* directions, and the simulation domain size was 2 µm × 2 µm × 600 nm. Minimum mesh sizes of 5, 5, and 2 nm were set along the *x*, *y*, and *z* directions, respectively. Random speckles resulting from scattering due to impurities, surface roughness, and grain boundaries on the bottom surface of the OHM were generated using 100 randomly oriented point dipole sources with a wavelength of *λ* = 488 nm and random initial phases at the substrate bottom within an area of 1 µm × 1 µm; therefore, the average distance between two adjacent dipoles was ≈100 nm. The electromagnetic waves generated from these randomly polarized dipole sources pass through the OHM layer (180 nm in thickness), interfering with each other to produce high spatial‐frequency speckle patterns on the top surface of the OHM. The cut‐off spatial frequency of the speckle patterns was determined using the fast Fourier transform in the spatial‐frequency domain.

## Conflict of Interest

The authors declare no conflict of interest.

## Supporting information

Supporting Information

## Data Availability

The data that support the findings of this study are available from the corresponding author upon reasonable request.
