# Supplementary material for: Metamaterial‐Assisted Illumination Nanoscopy with Exceptional Axial Resolution
Source: Adv Sci (Weinh). 2024 Aug 20;11(39):2404883. doi: 10.1002/advs.202404883 (PMC11497044; doi:10.1002/advs.202404883)
Supplement: Supplementary file 1 — Supporting Information [file ADVS-11-2404883-s001.docx]

**Supporting Information**

Metamaterial-assisted illumination nanoscopy with exceptional axial resolution

Yeon Ui Lee^†^, Shilong Li^†^, Junxiang Zhao, Clara Posner, Jin Zhang, Zhaowei Liu^*^

^†^These authors contributed equally to this work.

^*^Email: zhaowei@ucsd.edu

**Contents**

**S1. High spatial frequency illumination**

**S2. Cos-7 cell transfection**

**S3. Distance-dependent photobleaching dynamics**

**S4. Numerical study of 3D MAIN**

**S5. Overview of 3D super-resolution microscopy methods**

**S1.** **High spatial frequency illumination**

The basic physical principle of the proposed 3D MAIN is the spatial frequency mixing^[1,2]^: The maximum spatial frequency achievable by a far-field fluorescence microscopy with a non-uniform illumination is $f_{\det}+f_{\mathrm{ill}}$, where $f_{\det}={2n}/{\lambda_{\det}}$ ($f_{\mathrm{ill}}={2n}/{\lambda_{\mathrm{ill}}}$) is the detection (illumination) spatial frequency, $\lambda_{\det}$ ($\lambda_{\mathrm{ill}}$) is the detection (illumination) wavelength, and $n$ is the refractive index of surrounding medium; one can thus extend the resultant imaging resolution to arbitrarily high values by using a high spatial frequency illumination. Figure S1A (S1B) shows the projected dispersion of optical modes supported by the OHM (a glass) used in this work; owing to the hyperbolic topology of the OHM, optical modes with extremely large wavevector components $k$ ($k=2\pi f$) along both the axial and lateral directions are available^[3]^, which can thus be used as the high spatial frequency illumination $f_{\mathrm{ill}}$ to realize 3D super-resolution imaging.


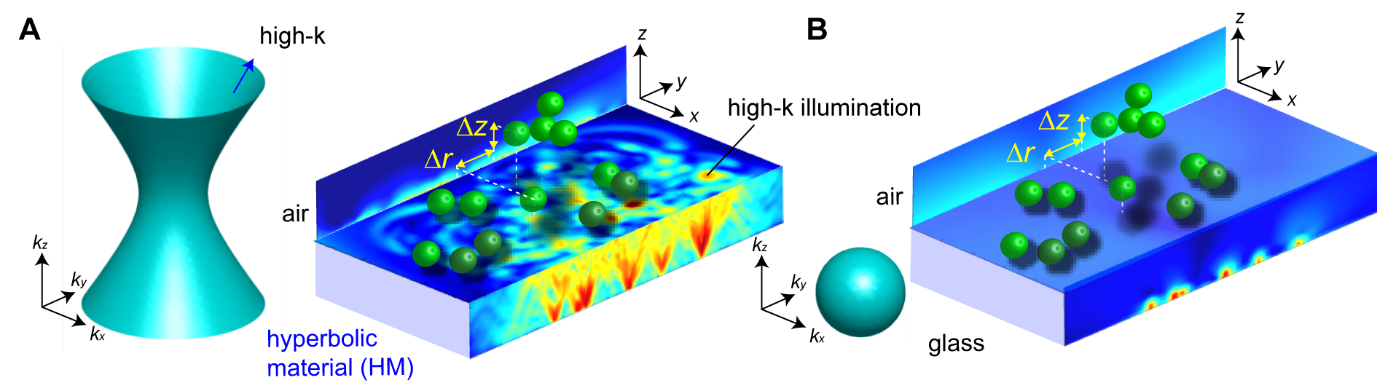


**Fig. S1 | High spatial frequency illumination enabled by OHMs.** (**A**) Randomly distributed near-field high-*k* speckles generated in the OHM transfer the fine structural information of fluorophores—e.g. the lateral ($\Delta r$) and axial ($\Delta z$) separations of two fluorophores—into the far-field microscopy. (**B**) Speckle field intensity distribution on top of a glass substrate is given for comparison.

**S2.** **Cos-7 cell transfection**

Cos7 (ATCC® CRL-1651) cells were cultured in Dulbecco modiﬁed Eagle medium (DMEM, Gibco) containing 4.5 g/L glucose and supplemented with 10% (v/v) fetal bovine serum (FBS, Sigma) and 1% (v/v) penicillin-streptomycin (Pen-Strep, Sigma-Aldrich). All cells were maintained in a humidiﬁed incubator at 37 °C under a 5%-CO_2_ atmosphere. 24 hours prior to the transfection, cells were seeded onto OHM substrates and grown to 50–70% conﬂuence. Cells were then transfected with 100-ng pcDNA3-Lifeact-Venus (Addgene plasmid #87613) for actin labeling or 100 ng of pcDNA3-Venus-CAAX (87612, Addgene) for plasma membrane labeling using Lipofectamine 2000 (Invitrogen) and grown an additional 24 hours before ﬁxation. Cells were washed with Phosphate-buffered saline (PBS) before ﬁxation with 4% paraformaldehyde and 0.2% glutaraldehyde PBS for 10 minutes at room temperature. Cells were quickly rinsed in PBS after ﬁxation and quenched with freshly made 0.1% NaBH4 ice-cold PBS. After quenching, cells were washed three times for 5 minutes each with PBS on a shaker. Cells were imaged at room temperature and stored in PBS at 4 °C.

**S3.** **Distance-dependent photobleaching dynamics**

Figure S2A summarizes the measured photobleaching results. Compared to the case of the glass, the photobleaching process of the fluorophores is significantly suppressed with the OHM. The relative photobleaching rate $\gamma$ is used to quantify this photobleaching suppression process, which is the ratio of the photobleaching decay rate ($\gamma_{\mathrm{OHM}}$) when the fluorophore is on the OHM to the case of the glass ($\gamma_{\mathrm{glass}}$):

$\gamma\equiv\frac{\gamma_{\mathrm{glass}}}{\gamma_{\mathrm{OHM}}}=\frac{F_{\mathrm{glass}}}{F_{\mathrm{OHM}}}=\frac{\tau}{\tau_{0}}$, (S1)

where $F_{\mathrm{OHM}}$ ($F_{\mathrm{glass}}$) is the Purcell factor when a fluorophore is on the OHM (glass), and $\tau=1/{\gamma_{\mathrm{OHM}}}$ ($\tau_{0}=1/{\gamma_{\mathrm{glass}}}$) is the photobleaching lifetime of the fluorophore on the OHM (glass). Note that the inverse proportion relationship between the photobleaching decay rate and the Purcell factor has been experimentally demonstrated^[4,5]^. To obtain the relationship between the $\gamma$ and the distance *d*, FDTD simulations (Figure S3) were performed for calculating the Purcell factor as the function of the distance. In these FDTD simulations, the fluorophore orientation was modeled as an average of 2/3 horizontally-polarized dipoles and 1/3 vertically-polarized dipoles (Figure S3A,B). Figure S2B shows the results of the distance-dependent Purcell factor. As can be clearly seen, there is a one-to-one relationship between the Purcell factor and the distance *d* and thereby the $\gamma$ and the distance *d,* according to Equation S1. The distance dependence of the $\gamma$ is summarized in Figure S2C. Since the $\gamma$ is also proportional to $\tau$, the axial (distance) information can be retrieved using $\tau$ by fitting the photobleaching decay curve. The distance-dependent curve is determined by the bulk permittivity and surface roughness, both affecting the Purcell factor. Previous studies have addressed material optimization^[6]^ and surface modifications^[4]^, and future work will focus on the biological and chemical factors that affect these two optical properties in specific applications.

In general, multi-exponential functions are used for the fitting. However, in most cases in this work, a bi-exponential fitting is applied, i.e. $y=A_{1}\exp(-t/\tau_{1})+A_{2}\exp\left( -t/\tau_{2} \right)$. Two fitted values $\tau_{1}$ and $\tau_{2}$ are obtained. One of the two values represents the fastest decay component, exhibiting a relative photobleaching rate of around 0.95, similar to that in the case of the glass (see Figure S2C). This component serves the purpose of reducing background signal interference, which is especially crucial when fluorophores are situated far (more than 50 nm) from the substrate. The second fitted value is used to calculate the distance *d* based on the one-to-one relationship shown in Figure S2C. During the intensity decay fitting, the fitting accuracy is confirmed via the standard errors of the mean (s.e.m., $\sigma$)^[5]^. In certain areas of the distance image, the standard errors of the mean $\sigma$ exceed 3 nm, indicating a low fitting accuracy; in this case, a tri-exponential fitting is applied: one fitted value is dedicated to background signal reduction, as mentioned before, while the other two values are used to map the distances of two spatially overlapped fluorophores.


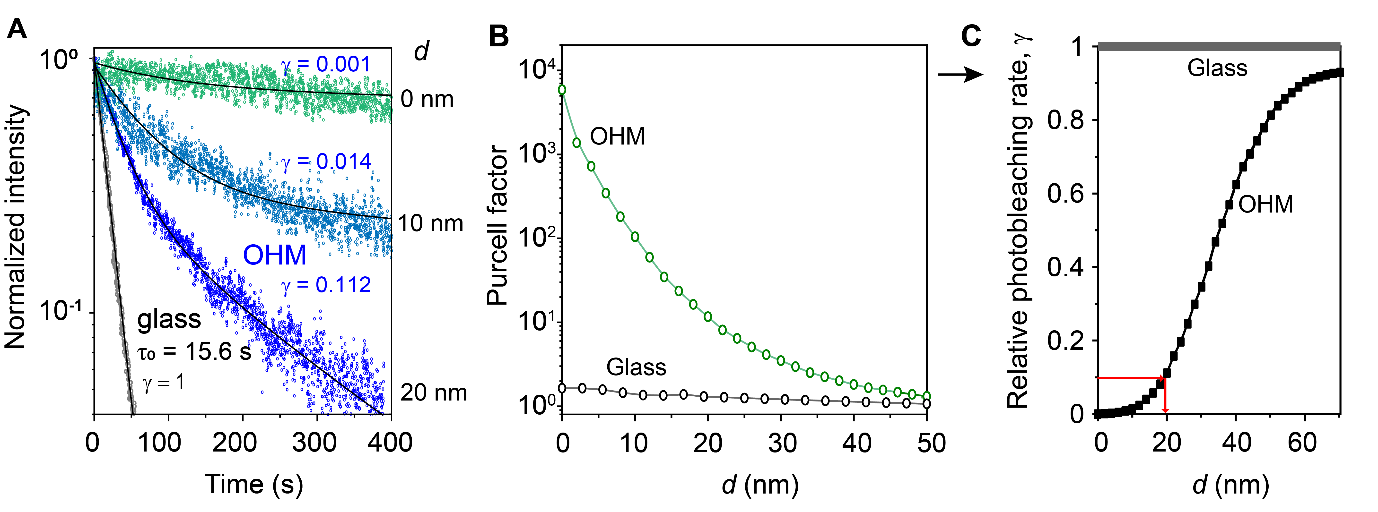


**Fig. S2 | Distance-dependent photobleaching dynamics.** (**A**) Photobleaching decay curves for fluorophores at different distances to the OHM surface. The result on top of the glass is also given. (**B**) Calculated distance-dependent Purcell factor of fluorophores on top of the OHM and glass substrates, respectively. (**C**) Relative photobleaching rate $\gamma$ as a function of the distance using the calculated Purcell factor (B) and the experimental data (A) based on Equation (S1).


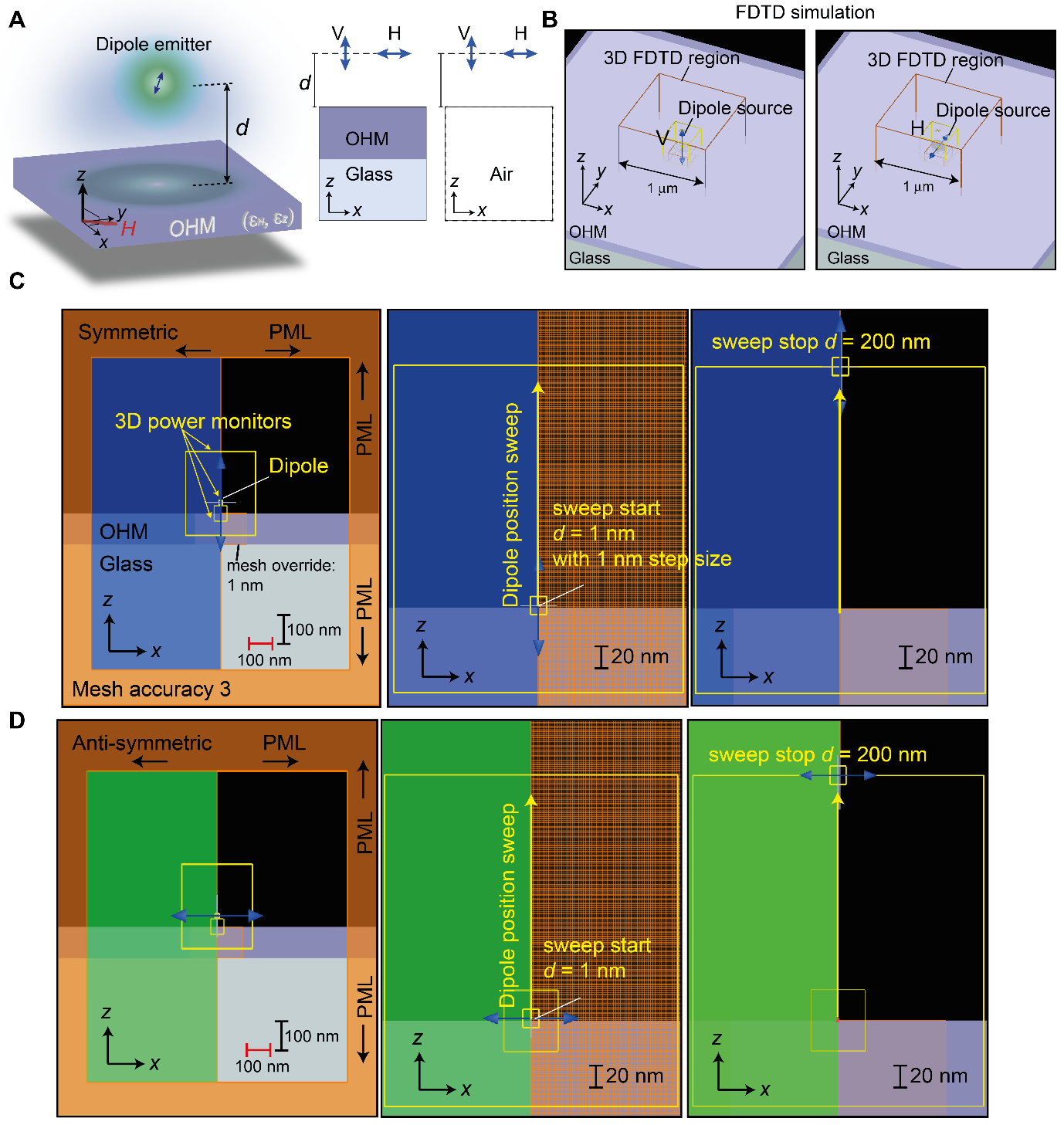


**Fig. S3 | FDTD simulations for calculating Purcell factor.** (**A**,**B**) Definition of the Purcell factor, which corresponds to the emission rate (and therefore power) enhancement of an emitter caused by the nearby OHM substrate. Using 3D power monitors in FDTD simulations, the power $P_{\mathrm{OHM}}$ emitted from a dipole source (the emitter) on top of the OHM substrate, and the power $P_{\mathrm{air}}$ emitted from the dipole in the absence of the OHM were obtained, and then the Purcell factor $F_{\mathrm{OHM}}={P_{\mathrm{OHM}}}/{P_{\mathrm{air}}}$. Since the dipole orientation within the cell is assumed to be random, the dipole for the Purcell factor calculation was modeled as an average of 1/3 vertically-polarized dipoles (V) and 2/3 horizontally-polarized dipoles (H). (**C**,**D**) Simulation configuration for calculating the distance-dependent Purcell factor. The distance *z* = *d* of the dipole source to the OHM surface was swept from 2 nm to 200 nm with a 1-nm step along the *z* direction at (*x*,*y*) = (0,0). A minimum mesh size of 0.25 nm was defined, and an additional overridden 1-nm mesh along the *z* direction was used. The perfectly matched layer (PML) boundary condition (BC) was adopted for the +*z* and –*z* directions. Depending on the structural symmetries, *y*-min BC = Symmetric, *y*-max BC = PML, *x*-min BC = Symmetric, and *x*-max BC = PML were used in (C), while *y*-min BC = Symmetric, *y*-max BC = PML, *x*-min BC = Anti-symmetric, *x*-max BC = PML were used in (D).

**S4.** **Numerical study of 3D MAIN**

Numerical studies on the implementation and performance of the proposed 3D MAIN were carried out using simulated fluorescent samples (see Figure S4). The fluorescent sample $\rho\left( x,y \right)$, such as that shown in the left panel of Figure S4A, was assigned with a time $t$ and distance $z$ dependent emission intensity $I_{xy}\left( t,z \right)=Ae^{{-t}/{\tau\left( z \right)}}$ (the right panel of Figure S4A) for each pixel $\left( x,y \right)$ to mimic the photobleaching dynamics of fluorophores, where $A$ is the initial intensity and $\tau\left( z \right)$ is the distance-dependent photobleaching lifetime. It was illuminated by $N\times L$ randomly developed speckles ($S_{i}$), and then convolved by the microscope detection PSF ($h$), producing $N\times L$ diffraction-limited images, as shown in Figure S4B. Now, following the procedures outlined above—i.e. applying the MAIN first and then the photobleaching lifetime fitting, a 3D super-resolution image was reconstructed, as shown in Figure S4C,D, which clearly reproduced the given fluorescent sample.


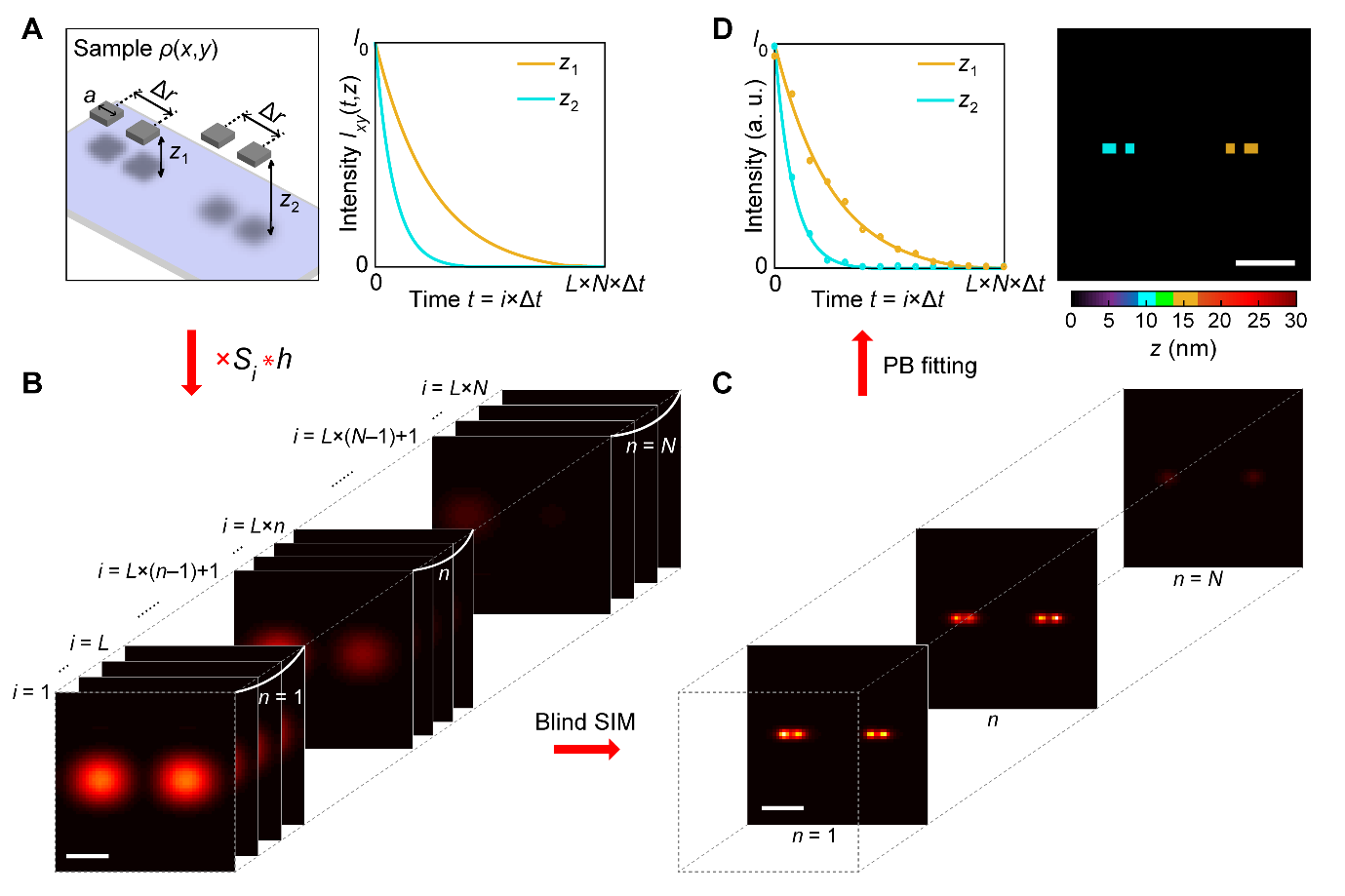


**Fig. S4 | Numerical study of 3D MAIN.** (**A**) Fluorescent sample consisting of two nearby nanoparticle pairs (size $a=15$ nm, separation $\Delta r=30$ nm) at two different distances ($z_{1}=10$ nm and $z_{2}=15$ nm) to the OHM surface (left panel, not to scale). The right panel shows the corresponding distance-dependent photobleaching curves. (**B**) Calculated $N\times L$ diffraction-limited images by multiplying ($\times$) the fluorescent sample by $N\times L$ speckles $S_{i}$ with the maximum spatial frequency $f_{ill,max}$ and then convolving ($*$) the results with the detection PSF $h$. Here, $L=100$, $N=14$, the integration time $\Delta t=0.5$ s, $f_{ill,max}=20f_{0}$, $f_{0}={2n}/{\lambda_{\mathrm{ill}}}$, $n=1.5$, $\lambda_{\mathrm{ill}}=470$ nm, $\lambda_{\det}=520$ nm were used. (**C**) $N$ reconstructed lateral super-resolution images from every $L$ successively diffraction-limited images using the Blind-SIM algorithm. Owing to the photobleaching, the sample intensity dropped. (**D**) Reconstructed 3D super-resolution image (right panel) with the axial super-resolution information obtained by fitting the dropped intensity curve (PB fitting, left panel). Scale bars: 100 nm.

**S5. Overview of 3D super-resolution microscopy methods**

**Table S1 | Overview of 3D super-resolution microscopy methods.** Note that the values in this table are not the maximum values reported in any specific article, but rather the typical values that these methods can achieve in most cases.

| Methods | Lateral  resolution | Axial  accuracy | Fluorophore  applicability | System  complexity | Phototoxicity | Field of view | Sectioning  ability | Refs |
| --- | --- | --- | --- | --- | --- | --- | --- | --- |
| 3D PALM/STORM | ~20 nm | ~50 nm | Photoactivatable/photoswitchable fluorophores | High | Middle | Narrow | Middle | ^[7–10]^ |
| 3D STED/GSD | ~20 nm | ~50 nm | Photostable fluorophores | High | High | Narrow | Middle | ^[11–14]^ |
| 3D SIM | ~100 nm | ~300 nm | Any fluorescent molecule | High | Low | Wide | Low | ^[15–17]^ |
| 3D MAIN | ~40 nm | ~5 nm | Any fluorescent molecule | Low | Low | Wide | High (Near-field excitation) | This work |

**References**

[1] R. Heintzmann, M. G. L. Gustafsson, *Nat. Photonics* **2009**, *3*, 362.

[2] F. Ströhl, C. F. Kaminski, *Optica* **2016**, *3*, 667.

[3] Y. U. Lee, C. Posner, Z. Nie, J. Zhao, S. Li, S. E. Bopp, G. B. M. Wisna, J. Ha, C. Song, J. Zhang, S. Yang, X. Zhang, Z. Liu, *Adv. Sci.* **2021**, *8*, 2102230.

[4] Y. U. Lee, S. Li, S. E. Bopp, J. Zhao, Z. Nie, C. Posner, S. Yang, X. Zhang, J. Zhang, Z. Liu, *Adv. Mater.* **2021**, *33*, 2006496.

[5] Y. U. Lee, J. Zhao, G. C. H. H. Mo, S. Li, G. Li, Q. Ma, Q. Yang, R. Lal, J. Zhang, Z. Liu, *Nano Lett.* **2020**, *20*, 6038.

[6] Y. U. Lee, K. Yim, S. E. Bopp, J. Zhao, Z. Liu, *Adv. Mater.* **2020**, *32*, 2002387.

[7] E. Betzig, G. H. Patterson, R. Sougrat, O. W. Lindwasser, S. Olenych, J. S. Bonifacino, M. W. Davidson, J. Lippincott-Schwartz, H. F. Hess, *Science* **2006**, *313*, 1642.

[8] S. A. Jones, S.-H. Shim, J. He, X. Zhuang, *Nat. Methods* **2011**, *8*, 499.

[9] Y. U. Lee, G. B. M. Wisna, S.-W. Hsu, J. Zhao, M. Lei, S. Li, A. R. Tao, Z. Liu, *ACS Nano* **2020**, *14*, 7666.

[10] M. J. Rust, M. Bates, X. Zhuang, *Nat. Methods* **2006**, *3*, 793.

[11] S. Bretschneider, C. Eggeling, S. W. Hell, *Phys. Rev. Lett.* **2007**, *98*, 218103.

[12] L. Schermelleh, A. Ferrand, T. Huser, C. Eggeling, M. Sauer, O. Biehlmaier, G. P. C. Drummen, *Nat. Cell Biol.* **2019**, *21*, 72.

[13] S. J. Sahl, S. W. Hell, S. Jakobs, *Nat. Rev. Mol. Cell Biol.* **2017**, *18*, 685.

[14] S. W. Hell, J. Wichmann, *Opt. Lett.* **1994**, *19*, 780.

[15] M. G. Gustafsson, *J. Microsc.* **2000**, *198*, 82.

[16] X. Li, Y. Wu, Y. Su, I. Rey-Suarez, C. Matthaeus, T. B. Updegrove, Z. Wei, L. Zhang, H. Sasaki, Y. Li, M. Guo, J. P. Giannini, H. D. Vishwasrao, J. Chen, S.-J. J. Lee, L. Shao, H. Liu, K. S. Ramamurthi, J. W. Taraska, A. Upadhyaya, P. La Riviere, H. Shroff, *Nat. Biotechnol.* **2023**, *41*, 1307.

[17] M. G. L. Gustafsson, L. Shao, P. M. Carlton, C. J. R. Wang, I. N. Golubovskaya, W. Z. Cande, D. A. Agard, J. W. Sedat, *Biophys. J.* **2008**, *94*, 4957.
